# Supplementary material for: Causal interplay between lactose intolerance and gut microbiota: a combined bidirectional Mendelian randomization and in vivo validation study
Source: Front Nutr. 2026 Jun 1;13:1803337. doi: 10.3389/fnut.2026.1803337 (PMC13265576; doi:10.3389/fnut.2026.1803337)
Supplement: Supplementary file 1 [file Data_Sheet_1.zip › supplementary materials/Forward/forest plot/ebi-a-GCST90027681.finngen_R12_E4_LACTONAS.pdf]

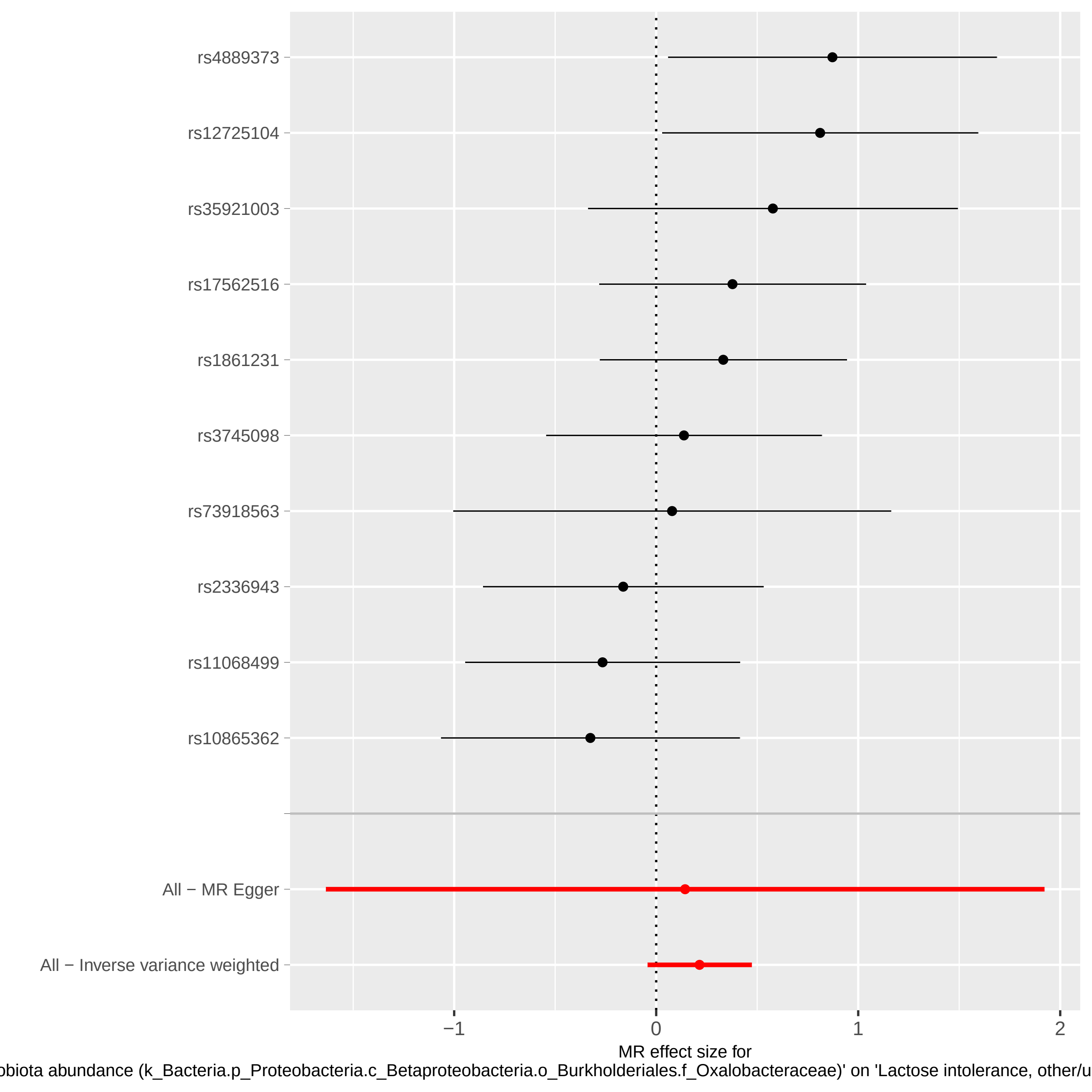

rs4889373
rs12725104
rs35921003
rs17562516
rs1861231
rs3745098
rs73918563
rs2336943
rs11068499
rs10865362
All − MR Egger
All − Inverse variance weighted
−1
0
1
2
MR effect size for
obiota abundance (k_Bacteria.p_Proteobacteria.c_Betaproteobacteria.o_Burkholderiales.f_Oxalobacteraceae)' on 'Lactose intolerance, other/u
